# Cancer stem cell-specific expression profiles reveal emerging bladder cancer biomarkers and identify circRNA_103809 as an important regulator in bladder cancer

**DOI:** 10.18632/aging.102816

**Published:** 2020-02-17

**Authors:** Tao Tao, Simin Yuan, Jinjian Liu, Da Shi, Mian Peng, Chong Li, Song Wu

**Affiliations:** 1 Department of Urology, The 3rd Affiliated Hospital of Shenzhen University, Shenzhen University, Shenzhen 518000, China; 2 Core Facility for Protein Research, Institute of Biophysics, Chinese Academy of Sciences, Beijing 100101, China; 3 Guangzhou Medical University, Guangzhou 510000, China; 4 Medical College, Shenzhen University, Shenzhen 518000, China; 5 Medical College, Anhui University of Science and Technology, Huainan 232001, China; 6 Department of Critical Care Medicine, The 3rd Affiliated Hospital of Shenzhen University, Shenzhen University, Shenzhen 518000, China; 7 Beijing Jianlan Institute of Medicine, Beijing 100190, China

**Keywords:** bladder cancer, stem cell, circRNA_103809

## Abstract

Bladder cancer stem cells (BCSCs), exhibiting self-renewal and differentiation capacities, may contribute to the tumor initiation, metastasis, recurrence and drug resistance of bladder cancer. However, the underlying functional mechanisms of BCSCs remain to be clarified. In this study, we describe the differentially-expressed mRNAs, lncRNAs, and circRNAs in BCSCs compared with that in bladder cancer non-stem cells (BCNSCs) through the transcriptome microarray data analysis using bladder cancer patients’ specimens. CircRNA_103809, the top one among the highly expressed circRNA identified in BCSCs, promotes the self-renewal, migration and invasion capabilities of bladder cancer by acting as a miR-511 sponge. Additionally, GO and KEGG pathway analysis suggest the differentially expressed genes identified may be involved in the cellular metabolism, differentiation and metastasis regulation of the cancer cells. Co-expression networks of lncRNAs/mRNAs and circRNAs/mRNAs constructed by WGCNA give a picture of the non-coding/coding RNAs regulating patterns in BCSCs. Notably, as core genes in the networks, AHCY, C6orf136 and LRIG1 show high potential to be prognosticators for bladder cancer. Therefore, further studies of non-coding RNA functional mechanisms in BCSCs is valuable for detecting the pathogenic mechanisms and discovering novel biomarkers in bladder cancer.

## INTRODUCTION

Bladder cancer (BC) is one of the most life-threatening malignancies with high morbidity and mortality rates worldwide [1]. Benefit from the continual development of neoplasm diagnosis and therapy methods, the death rate of cancer had been decreased in the past 10 years. However, because of the lack of progress in the treatment for bladder cancer these years, the death rate of bladder cancer had barely changed [2]. Deeper researches on the pathogenesis and molecular biology of bladder cancer are needed to improve the diagnosis and treatment methods. Recent studies have suggested the critical role of cancer stem cells (CSCs) or cancer-initiating cells in tumorigenesis of many cancers [3, 4], including bladder cancer [5, 6]. The CSCs display the capacities of self-renewal, differentiation and chemoresistance, which could affect the progression and recurrence of cancer significantly [7]. However, the molecular mechanisms underlying the stemness-like functions of bladder cancer stem cells (BCSCs), especially the genetic and epigenetic characters, remain largely elusive.

Two types of RNA without protein-coding potentials, termed as long non-coding RNA (lncRNA) and circular RNA (circRNA), were identified [8, 9] and proved to play important roles in many diseases recently [10–12]. LncRNAs are non-coding transcripts longer than 200 nucleotides [13]. Differentially expressed lncRNAs contribute to the invasiveness and metastasis of breast cancer [14], lung cancer [15] and colorectal cancer [16] by cis- and/or trans-regulating on their target genes’ expression [17, 18] and functioning as microRNA (miRNA) sponges [19, 20]. LncRNAs also participate in disease-related biological processes like cell proliferation, cell motility, and immunity, thus contribute to the inflammation response from diseases like cardiovascular disorders, and autoimmune diseases [21–23]. On the other hand, roles of circRNAs in diseases remains largely unknown. Previous studies have shown that circRNAs could work as miRNA sponges and consequently repress their function too [24–26]. When some circRNAs were upregulated aberrantly, the expression of miRNA targets, like some cytokines [27], would be increased and became potential oncogenes by promoting cell cycles and inhibiting cell apoptosis [28]. Additionally, lncRNAs and circRNAs are considered as effective biomarkers for diagnosis and prognosis in some diseases [29, 30]. These findings of lncRNAs and circRNAs in diseases revealed that the expression features as well as potential functions of these RNAs in BCSCs, which are still unclear, are worth to be investigated further.

In this study, we represented the expression profiles and interactive networks of mRNAs, lncRNAs, and circRNAs associated with BCSCs for the first time. Based on the RNAs expression data arose from two microarrays of three bladder cancer specimens from patients, differentially expressed circRNAs, lncRNAs and mRNAs in BCSCs when compared to that in BCNSCS were profiled. Then the most highly expressed circRNA in BCSCs named circRNA_103809 was chosen to be demonstrated further about its functions. GO and KEGG pathway enrichment analyses were performed to investigate the biological pathways tuned by these differentially expressed RNAs. The Co-expression networks were constructed to display the potential important interactive network within BCSCs. The expression of some of the core genes in the co-expression networks were found to be correlated to the prognosis of BC patients. Results from this study highlighted the biological processes regulated by non-coding RNAs in BCSCs and provided inspiring clues about future research for novel diagnostic and therapeutic targets based on non-coding RNAs in bladder cancer.

## RESULTS

### Differential expression profiles of mRNAs, lncRNAs and circRNAs in BCSCs

**Figure 1:**
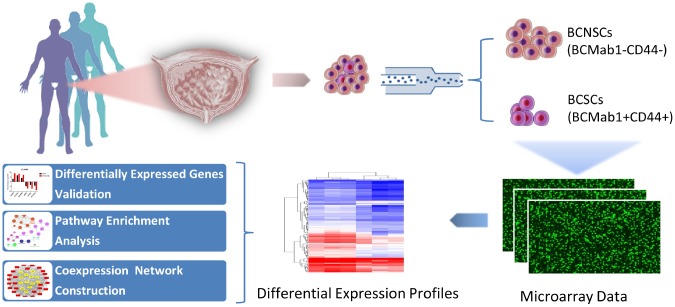
**Research workflow of mRNAs, lncRNAs and circRNAs analysis in bladder cancer stem cells.**

**Figure 2:**
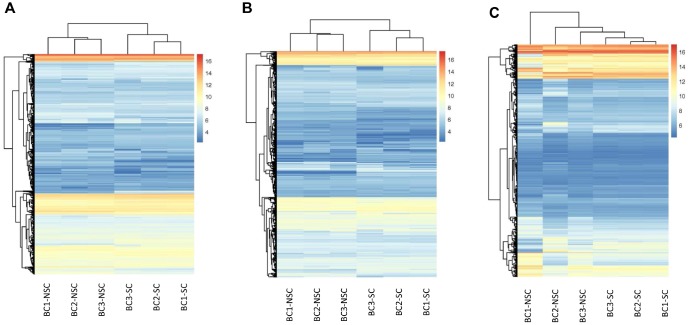
**Hierarchical Clustering of all Expressed mRNAs, lncRNAs, circRNAs.** Unsupervised hierarchical clustering of expressed mRNAs (**A**), lncRNAs (**B**), and circRNAs (**C**) from 3 pairs (BCSC vs BCNSC) of bladder cancer samples. Both expression data and sample clustering were done using average linkage and uncentered pearson correlation metric by Cluster 3.0, and results were visualized by TreeView.

**Figure 3:**
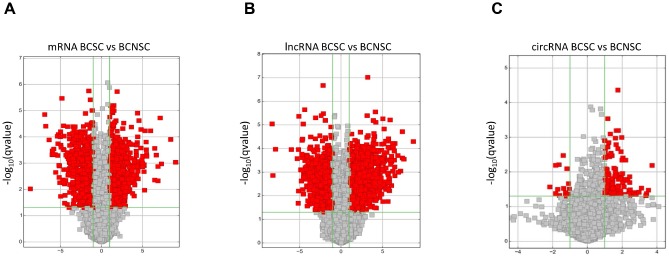
Differentially expressed mRNAs (**A**), lncRNAs (**B**) and circRNAs (**C**) between BCSCs and BCNSCs were analyzed. Volcano plot of the p-values as a function of fold change for mRNAs, lncRNAs and circRNAs indicate the differentially expressed genes between the BCSCs and BCNSCs. Grey dots represent RNAs not significantly differentially expressed (P-value > 0.05) and the red dots represent RNAs differentially expressed (P-value < 0.05).

**Figure 4:**
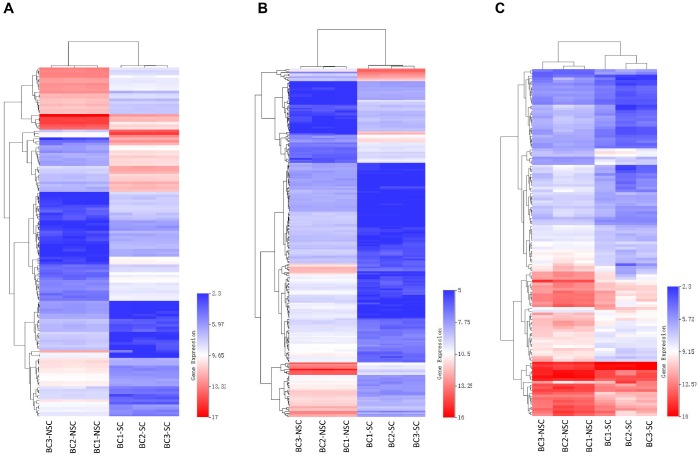
Heatmap of differentially expressed mRNAs (**A**), lncRNAs (**B**) and circRNAs (**C**) were represented. Red through blue color indicates high to low expression level. Each row indicates one transcript, and each column represents one sample.

Our previous studies indicated that the monoclonal antibody BCMab1 recognized aberrantly glycosylated integrin α3 and could be used in the isolation of human bladder cancer stem cells (BCSCs) when combined with CD44 [31]. To identify genes associated with functions of BCSCs, we performed a transcriptome microarray analysis of human BCSCs (BCMab1+CD44+) and BCNSCs (BCMab1-CD44-) isolated from three BC patients. Workflow of this study was shown in Figure 1. The expression matrices for 3 pairs of BCSCs and BCNSCs samples were constructed after data preprocessing. Heatmaps for the expression of total mRNAs, lncRNAs and circRNAs were shown in Figure 2 respectively. Volcano plots were used for assessing gene expression variation between BCSCs and BCNSCs (Figure 3). A total of 2849 mRNAs, 2698 lncRNAs and 127 circRNAs were found to be differentially expressed with the P-value < 0.05 and fold change > 2.0. Compared with that in BCNSCs, 1685 mRNAs, 1309 lncRNAs and 113 circRNAs were highly expressed, while 1164 mRNAs, 1389 lncRNAs and 14 circRNAs were lowly expressed in BCSCs. Hierarchical clustering analysis showed systematic variations in the expression of these RNAs among samples (Figure 4), and suggested that the expression of mRNAs, lncRNAs and circRNAs in BCSCs differ from those in BCNSCs.

### Structure feature and chromosomes distribution of BC expressing non-coding RNAs

**Figure 5:**
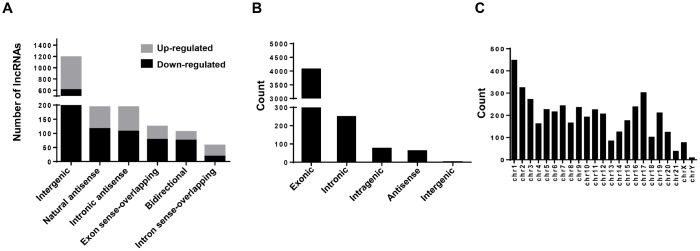
**Character analysis of differentially expressed lncRNAs and circRNAs.** (**A**) Differentially expressed lncRNAs (BCSCs vs BCNSCs) were classified into 6 categories according to the genomic loci of their neighboring genes. The grey portion and black portion of the column represents up-regulated and down-regulated lncRNAs in BCSCs respectively. (**B**) Differentially expressed circRNAs (BCSCs vs BCNSCs) were classified into 5 categories according to the genomic loci of their neighboring genes. (**C**) Counts of differentially expressed circRNAs were classified according to their loci on human chromosomes.

In this study, a total of 12,261 annotated lncRNAs and 4,451 novel circRNAs were identified from the BC samples. To present the structural features of these two kinds of non-coding transcripts in BC cells, analysis of gene structure and sequence conservation on the lncRNAs and circRNAs detected in this microarray assay was conducted. Our results showed that intergenic and natural antisense lncRNAs constitute the largest number in all expressing lncRNAs, respectively (Figure 5A). And exonic and intronic circRNAs constitute the largest number in all expressing circRNAs (Figure 5B). In addition, analysis of the distribution of circRNAs on 24 chromosomes (22 autosomes and 2 sex chromosomes) showed that circRNAs expressed in bladder cancer cells are mainly distributed on the chr1 and the chr17 (Figure 5C).

### Validation of differentially expressed mRNAs, lncRNAs and circRNAs

**Figure 6:**
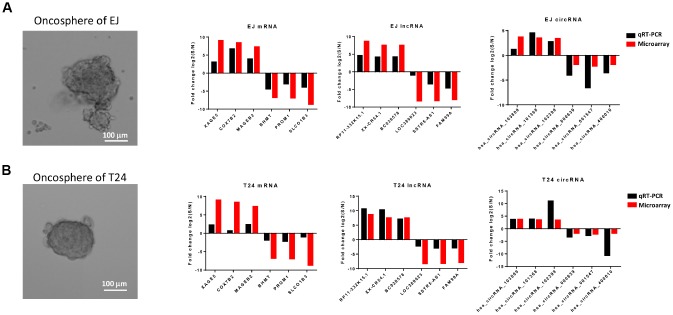
**Comparison of results from microarray and qRT-PCR.** qRT-PCR results of 6 mRNAs (XAGE5, COX7B2, MAGEB2, BHMT, PROM1 and SLCO1B3), 6 lncRNAs (RP11-332K15.1, XX-CR54.1, BC038578, LOC389023, SSTR5-AS1, and FAM99A), and 6 circRNAs (hsa_circRNA_103809, hsa_cirRNA_101368, hsa_cirRNA_102399, hsa_cirRNA_000639, hsa_cirRNA_001547, and hsa_cirRNA_400010) from the oncosphere formed bladder cancer cell line EJ (**A**) and T24 (**B**) were compared with the expression data gained from the microarray. The red column represents the fold change values obtained from microarray and the black column displays the fold change values of qRT-PCR.

To validate the differential expression profiles gained from the microarray analysis, six of mRNAs, lncRNAs and circRNAs were randomly selected respectively from the differentially expressed genes and be verified *in vitro*. According to previous reports, cells which are capable of forming suspensive oncospheres in a serum-free culture medium supplemented with selected growth factors show high stemness, oncosphere formation culture then be considered an efficient method to enrich cancer stem cells *in vitro* [32, 33]. Therefore, we enriched bladder cancer stem cells from two bladder cancer cell lines T24 and EJ by oncosphere formation culture (Figure 6A). Validation was performed by comparing the expression level of selected RNAs in oncosphere cells with that in non-sphere cells, and the trends differential expression were consistent with the results from microarray analysis based on patients’ samples (Figure 6B, 6C).

### circRNA_103809 serves as a sponge for the miR-511 and induces bladder cancer progression

**Figure 7:**
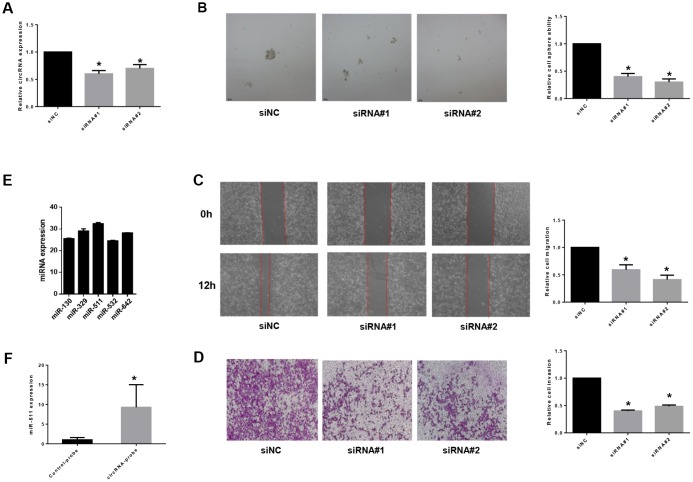
**circRNA_103809 induces bladder cancer progression by sponging miR-511.** (**A**) qRT-PCR analysis of the transfection efficiency of siRNA transfection in EJ cells. Under-expression of circRNA_103809 causes a diminished oncosphere (**B**), migration (**C**) and invasion (**D**) capacity in EJ cells. (**E**) qRT-PCR analysis of miR-130, miR329, miR511, miR532 and miR-642 expression in EJ cells. (**F**) EJ cell lysates were subject to RNA pull-down assay. miR-511 can be pulled down by circRNA_103809 probe.

To study functions of the differentially expressed non-coding RNAs identified in BCSCs, we chose circRNA_103809, the most variable gene among the highly expressed circular RNAs, to investigate further. CircRNA_103809 locates at chr5:32379220-32388780 (CircBase ID: hsa_circ_0072088), and was found derived from the ZFR gene and may be associated with tumor relapse of hepatocellular carcinoma [34]. In this study, we knocked down the expression of circRNA_103809 in bladder cancer cells with two siRNAs that cover the back-splicing region of circRNA_103809, and confirmed the efficiency by qRT-PCR (Figure 7A). It turned out that the silencing of circRNA_103809 significantly reduced the oncosphere formation, migration and invasion abilities of bladder cancer cells (Figure 7B–7D). These findings indicated that under-expression of circRNA_103809 may be capable of reducing the progression of BC. To address whether circRNA_103809 works as miRNA sponge in BC cells, we predicted the circRNA/miRNA interaction with Arraystar’s homemade miRNA target prediction software. According to the prediction, circRNA_103809 may interact with five miRNAs (Supplementary Figure 1). Then to validate whether circRNA_103809 serves as the binding platform of these miRNAs, we performed RNA pulldown assay in the EJ cells. As shown in Figure 7E, we first determined that miRNAs expression in EJ cell line. Then we used circRNA_103809 probe pull-down the endogenous different miRNAs, we found that only miR-511 was specifically enriched by circRNA_103809 in EJ cells (Figure 7F). All these experiments proved that circRNA_103809 may function as a sponge for miR-511 in BC progression.

### Biological processes and pathways enrichment of differentially expressed RNAs

The expression of many lncRNAs is significantly correlated with the functions of their neighboring protein-coding genes [35]. And the processing of circRNAs can affect the splicing of their precursor transcripts, leading to altered gene expression outcomes [36]. To clarified the biological processes influenced by the differentially expressed lncRNAs, circRNAs and mRNAs in BCSCs, we performed pathway and function enrichment analysis on gene sets constituted by neighboring protein-coding genes of the lncRNAs, precursor transcripts of the circRNAs, and the mRNAs respectively.

**Figure 8:**
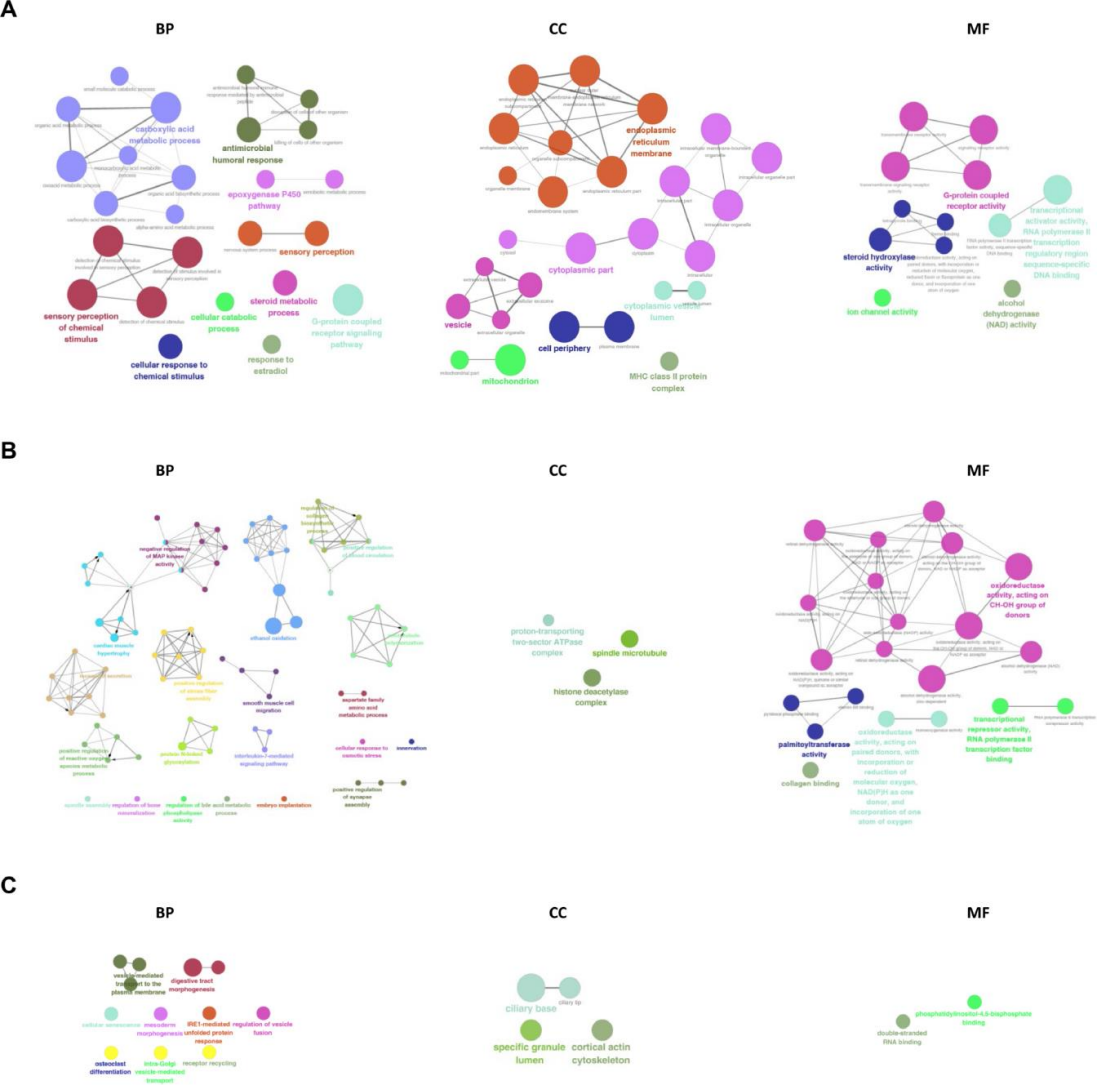
**GO analysis of differentially expressed mRNAs, lncRNAs, and circRNAs.** GO and pathway terms were connected and grouped based on their functional similarity, which is calculated using kappa statistics in ClueGO. Terms in the same group are specified by identical colors, and their size is proportional to their statistical significance. The most significant term in each group is highlighted by a colorful and bigger font.

The GO network and diagrams were obtained from the Gene Ontology enrichment analysis (Figure 8). According to the GO category of “Biological Process”, the most significant terms in the mRNA group were sensory perception of chemical stimulus, carboxylic acid metabolic process and G-protein coupled receptor signaling pathway. While in the lncRNA group, the most significant terms were ethanol oxidation, innervation, positive regulation of microtubule polymerization, interleukin-7-mediated signaling pathway, and positive regulation of vascular endothelial growth factor production. The most significant terms in the circRNA group were receptor recycling, digestive tract morphogenesis, intra-Golgi vesicle-mediated transport, Golgi to plasma membrane transport, prostate gland development, vesicle-mediated transport to the plasma membrane. In the GO category of “Cellular Component”, endoplasmic reticulum membrane, cytoplasmic part, vesicle, cell periphery and mitochondrion were the most significant in the mRNA group and the most significant terms in the lncRNA group were histone deacetylase complex, spindle microtubule, and proton-transporting two-sector ATPase complex. In the circRNA group terms were cortical actin cytoskeleton, specific granule lumen, ciliary tip, and ciliary base. In the GO category of “Molecular Function”, we recorded transcriptional activator activity, RNA polymerase II transcription regulatory region sequence-specific DNA binding, G-protein coupled receptor activity and steroid hydroxylase activity as the most representative terms in the mRNA group. The most representative terms in the lncRNA group were alcohol dehydrogenase activity, zinc-dependent, retinal dehydrogenase activity, alcohol dehydrogenase (NAD) activity, steroid dehydrogenase activity, acting on the CH-OH group of donors, NAD or NADP as acceptor, RNA polymerase II transcription corepressor activity. In the circRNA group terms were double-stranded RNA binding and phosphatidylinositol-4,5-bisphosphate binding.

**Figure 9:**
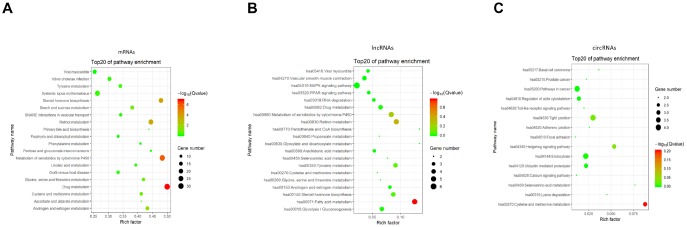
KEGG pathway enrichment of differentially expressed mRNAs (**A**), lncRNAs (**B**) and circRNAs (**C**). Enriched KEGG pathway scatter plot showing statistics of pathway enrichment in between the BCSCs and BCNSCs. The vertical axis represents the pathway name and the horizontal axis represents the rich factor. The size of the dot represents the number of differentially expressed genes in the pathway and the color of the point corresponds to the different q-value range.

As a major public database of pathways, the Kyoto Encyclopedia of Genes and Genomes has been used to determine the significantly enriched pathways for candidate target genes compared with the entire genome background [37, 38]. The KEGG analysis performed on the gene set mentioned above had shown the pathways associated with the differentially expressed mRNAs, lncRNAs, and circRNAs in BCSCs respectively (Figure 9). For mRNAs, the drug metabolism, metabolism of xenobiotics by cytochrome P450 and steroid hormone biosynthesis were identified as the top enriched KEGG pathways (Figure 9A). Fatty and metabolism, pantothenate and CoA biosynthesis, glyoxylate and dicarboxylate metabolism were the most significantly enriched pathways for lncRNAs (Figure 9B). Whereas, the significantly enriched pathways were cysteine and methionine metabolism, hedgehog signaling and tight junction for circRNAs (Figure 9C).

### LncRNA-mRNA and circRNA-mRNA co-expression networks in BCSCs

**Figure 10:**
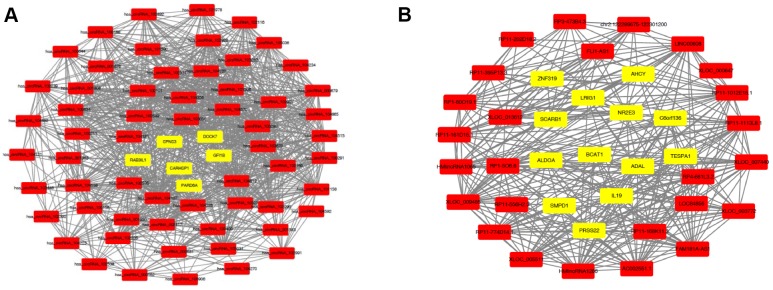
**Coexpression networks constructed by weighted correlation network analysis.** Different colors of dots were used to show different types of genes, with yellow for mRNAs and red for lncRNAs (**A**) or circRNAs (**B**).

**Figure 11:**
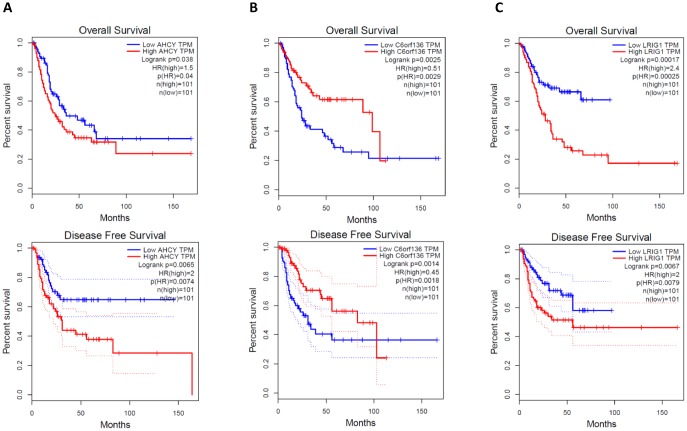
Overall survival and disease free survival analysis of AHCY (**A**), C6orf136 (**B**) and LRIG1 (**C**) with bladder cancer data from TCGA.

Generally, the functions of lncRNAs and circRNAs were mainly performed by interacting with their targets [8, 9]. The mRNAs whose expression is correlated with circRNAs or lncRNAs could be important targets of the latter. Here, co-expression networks of differentially expressed lncRNAs-mRNAs and circRNAs-mRNAs were constructed through weighted correlation network analysis (WGCNA). The coexpression network of mRNAs and circRNAs was comprised of 66 network nodes and 1088 connections, and this network included 6 mRNAs, CARHSP1, DOCK7, GFI1B, PARD6A, RAB3IL1 and SPNS3 constituting probably the core of the network (Figure 10A). Meanwhile, the coexpression network of mRNAs and lncRNAs was comprised of 39 network nodes and 380 connections, and this network included 13 mRNAs, ADAL, BCAT1, IL19, KIAA0748, NR2E3, PRSS22, SCARB1, SMPD1, ZNF319, ALDOA, AHCY, C6orf136 and LRIG1 (Figure 10B). Next, we analyzed the relationship between the expression of these core genes and the survival prognosis of patients in the TCGA bladder cancer database. A significant difference of overall survival and disease free survival between the high expression groups and the low expression groups for AHCY, C6orf136 and LRIG1 was observed (Figure 11, *P* < 0.05). Therefore, the expression levels of AHCY, C6orf136 and LRIG1 could be prognosticators of survival in BC patients.

## DISCUSSION

Bladder cancer is one of the most common cancers, which ranks the 4th among cancers in males according to the USA cancer statistics of 2017 [1]. Cancer stem cells with high tumorigenic, drug-resistant and metastatic characters were considered may contribute to the relapse and metastasis of cancer [39]. Making use of a mAb (BCMab1) against CD44^+^ human bladder cancer cells that recognize aberrantly glycosylated integrin α3β1, we isolated a subset of bladder cancer cells from primary samples in the previous study. We showed that the BCMab1^+^CD44^+^ cells act as bladder cancer stem cells (BCSCs) and are correlated with clinicalpathologic features of bladder cancer [31]. To gain some insights into biological functions of non-coding RNAs in the CSCs of bladder cancer, we performed a comprehensive analysis of microarray data of BCSCs and BCNSCs from three primary samples. We identified the top differentially expressed lncRNAs and circRNAs, then validated their expression by qRT-PCR. By constructing lncRNAs/mRNAs and circRNAs/mRNAs coexpression networks, we identified mRNAs which may function as core molecules in the genes regulation network of BCSCs. Results from overall survival analysis and disease free analysis with clinical data from TCGA revealed that core mRNAs in the coexpression networks, AHCY, C6orf136 and LRIG1, correlate with the progression and recurrence of bladder cancer significantly.

As shown in previous reports, AHCY (S-adenosylhomocysteine hydrolase) catalyzes the reversible hydrolysis of SAH (S-adenosylhomocysteine) to adenosine and l-homocysteine. This enzyme is frequently overexpressed in many tumor types and is considered to be a validated anti-tumor target [40]. Inhibition of AHCY decreased cell proliferation by G2/M arrest in MCF7 cells [41]. LRIG1 (Leucine-rich repeats and immunoglobulin-like domains 1) is a cell surface protein that antagonizes ERBB receptor signaling by downregulating receptor levels. Interestingly, a contrast to what we found in bladder cancer, LRIG1 was shown to be associated with good survival in 7 types of cancers such as breast cancer, uterine cervical cancer, and head-and neck cancer etc [42]. And it was also reported that LRIG1 marks stem cells in the gut and may maintain the intestinal epithelial homeostasis [43]. Lack of previous reports on the functions of C6orf136, further studies are needed to clarify cancer associated functions of this gene.

Whole-genome and whole-exome mapping have provided an overview of the genomic aberrance associated with the tumorigenesis of bladder cancer [44]. The expression profile data gained from microarray in this study improved the understanding of molecular mechanisms of bladder cancer stem cells by providing information about novel lncRNAs and circRNAs involved in the critical biological pathway of BCSCs.

To unravel the working mechanisms of these non-coding RNAs in the biological process regulation and prognosis of bladder cancer, this study has performed Gene Ontology (GO) and Kyoto Encyclopedia of Genes and Genomes (KEGG) pathway analysis on associated coding genes of lncRNAs and circRNAs which are differentially expressed between BCSCs and BCNSCs. The results from the analysis revealed that lncRNAs and circRNAs work through different pathways on the function maintenance of BCSCs. For example, lncRNAs may help to support the energy consumption during the efficient proliferation of bladder cancer stem cells by regulating the fatty acid metabolism process. And circRNAs tend to affect the metastasis ability and differentiation of bladder cancer cells by acting on the tight junction and hedgehog signaling associated pathway. The pathway mapping for mRNAs expressed differentially in the two types of cells was also performed and suggests that the drug metabolism function of BCSCs is quite different from BCNSCs, corresponding to the hypothesis that BCSCs may contribute to the chemoresistance of bladder cancer.

In summary, the interactive network of non-coding RNAs and mRNAs constructed by transcription data comparison between BCSCs and BCNSCs suggests that the RNA regulation network plays important role in stemness related functions of bladder cancer cells. Exploring further for critical non-coding RNAs working in BCSCs is required for a better understanding of the molecular mechanisms of bladder cancer recurrence and metastasis.

In conclusion, differentially expressed mRNAs, lncRNAs and circRNAs were screened from BCSCs and BCNSCs of three clinical bladder cancer specimens and analyzed. A total of 2849 mRNAs, 2698 lncRNAs and 127 circRNAs were differentially expressed, and regulate expression of their related genes and play important roles in the stemness maintaining of bladder cancer cells. The relevant signaling pathways of these RNAs shed light on the future study to investigate detailed mechanisms of bladder cancer initiation and progression. The current work also gives rise to the consideration that metabolism processes, especially those which are related to the drug metabolism should be investigated further to unravel the chemotherapy resistance mechanisms of bladder cancer stem cells.

## MATERIALS AND METHODS

### Clinical specimens and cell lines

The 3 samples of bladder tumor specimens used in this study were obtained from patients during operation. All human studies were reviewed and approved by the Institute of Biophysics, Chinese Academy of Sciences, and written informed consent was provided according to the World Medical Association Declaration of Helsinki. Bladder cancer cell lines EJ and T24 used in this study were obtained from ATCC, the American Type Culture Collection. Both cell lines were cultured with RPMI 1640 medium (Gibco, NY, USA) supplemented with 10% fetal bovine serum (FBS) and incubated at 37°C in a humidified atmosphere containing 5% CO_2_.

### Flow cytometry sorting cancer stem cells and microarray assay analysis

Primary human bladder cancer stem cells were sorted by flow cytometry (BD FACSAria II) as previously described [31]. Total RNAs from each sample was quantified using the NanoDrop ND-1000. The sample preparation and microarray hybridization was performed based on the Arraystar’s standard protocols. Briefly, total RNAs from each sample was amplified and transcribed into fluorescent cRNAs utilizing random primers according to Arraystar’s Super RNA Labeling protocol (Arraystar Inc.). The labeled cRNAs were hybridized onto the Arraystar Human circRNA Array (8×15K, Arraystar). After having washed the slides, the arrays were scanned by the Agilent Scanner G2505C.

Differentially expressed LncRNAs, circRNAs and mRNAs with statistical significance between the two groups of BCSCs and BCNSCs were identified through P-value/FDR filtering. Hierarchical Clustering and combined analysis were performed using homemade scripts.

### Oncosphere formation and quantitative real-time PCR

EJ and T24 cells were seeded in ultra-low attachment culture dishes (Corning) with Knock-Out DMEM/F-12 medium (Gibco, 12660012) supplemented with 20 ng/mL EGF (Invitrogen, PHG0314), 20 ng/mL bFGF (Invitrogen, 13256029), 1% N2 (Gibco, 17502048), and 2% B27(Gibco, 17504044), and incubated in a CO_2_ incubator for one to two weeks. Then the oncosphere cells were analyzed by qRT-PCR. Total cDNA of each sample was synthesized using two-step reverse transcriptase Kit (Vazyme Biotech Co., Ltd., Nanjing, China) according to the manufacturer’s instructions. qRT-PCR were performed using StepOnePlus Real-Time PCR System (Applied Biosystems, Carlsbad, CA, USA) and UltraSYBR Mixture (Qiagen, Shanghai, China). Each reaction (in 10 μL) contained 5 μL 2 × QuantiFast® SYBR® Green PCR Master Mix, 0.4 μL primers (5 μM), and 1 μL cDNA. The gene expression levels were normalized with the reference gene GAPDH by using 2^−ΔΔCt^ value methods. Primers sequences for the detected genes were listed in Supplementary Table 1.

### Cell migration and invasion assay

Cell migration was performed with *in vitro* scratch assay. The invasion was determined using modified Boyden chambers coated with Martrigel matrix in 24-well plate (BD Biosciences) as previously described [45].

### circRNA targets miRNA prediction

circRNA_103809-miRNA interaction was predicted with miRNA target prediction software based on TargetScan and miRanda (Arraystar’s home-made).

### RNA interference

siRNA duplexes were synthesized by GemaPharma (Shanghai, China) and the siRNA sequences were as below: circRNA_103809 siRNA#1, sense 5′-CCAAGCUGGCCCUUACGUCTT-3′ and anti-sense 5′-GACGUAAGGGCCAGCUUGGTT-3′; circRNA_103809 siRNA#2, sense 5′-GCUGGCCCUUACGUCGUCCTT-3′ and anti-sense 5′-GGACGACGUAAGGGCCAGCTT-3′.

### Biotin-labeled pull-down assay

Biotinylated circRNA_103809 (GenePharma, Shanghai, China) pull-down assay with target mRNAs was performed as described earlier [46].

### GO and KEGG enrichment analysis

To explore the function of mRNAs, lncRNAs and circRNAs, the Gene Ontology (GO) and Kyoko Encyclopedia of Genes and Genomes (KEGG) enrichment analysis were conducted. The ClueGO (v1.18.0) was used to perform the GO enrichment analysis of differentially expressed genes or target genes of lncRNAs or source genes of differentially expressed circRNAs, in which gene length bias was corrected. All three GO categories, namely cellular component, biological process, and molecular function, were included, and GO terms with the q-value < 0.05 were considered significantly enriched. R software was used to examine the statistical enrichment analysis of differential expression genes or lncRNA target genes or source genes of differentially expressed circRNAs in KEGG pathways. The enriched information was evaluated by the statistical test and correction. The EASE score was calculated to test the relevance, and p-value < 0.05 was considered significantly enriched by differentially expressed genes.

### Correlation and coexpression analysis

The coexpression analysis was based on weighted correlation network analysis (WGCNA). WGCNA package in R as a powerful tool was used to make and analyze a co-expression network of selected lncRNAs, circRNAs and mRNAs. Compared to general methods, such as Pearson’s correlation coefficient, WGCNA uses the soft threshold, which can provide more extensive and exact correlation between transcripts. Differentially expressed lncRNAs, circRNAs and mRNAs with fold change > 2, P < 0.05, and FDR < 0.05 were analyzed. The value of parameter soft threshold > 0.98 and P-value < 0.05 was recommended for the coexpression analysis. k-Core scoring was used to determine core transcripts of coexpression networks. A higher k-core score means a more central location of a transcript within a network. The soft threshold was adjusted to 0.8 to obtain the lncRNA and circRNA coexpressed mRNA cluster for further functional analysis of lncRNAs and circRNA.

### Statistical analysis

We used UALCAN analysis to estimate the effects of hub gene expression levels on patient survival in the Cancer Genome Atlas (TCGA) bladder cancer datasets. Available TCGA patient survival data were also used for Kaplan–Meier survival analyses [47]. The statistical difference in gene expression of qRT-PCR results was analyzed by Student’s t-test. It was considered to be statistically significant when p-value < 0.05.

## Supplementary Material

Supplementary Figure 1

Supplementary Table 1

## References

[1] Siegel RL, Miller KD, Jemal A. Cancer Statistics, 2017. CA Cancer J Clin. 2017; 67:7–30. 10.3322/caac.21387 28055103

[2] Soloway MS. Bladder cancer: lack of progress in bladder cancer—what are the obstacles? Nat Rev Urol. 2013; 10:5–6. 10.1038/nrurol.2012.219 23165404

[3] Reya T, Morrison SJ, Clarke MF, Weissman IL. Stem cells, cancer, and cancer stem cells. Nature. 2001; 414:105–11. 10.1038/35102167 11689955

[4] Vermeulen L, de Sousa e Melo F, Richel DJ, Medema JP. The developing cancer stem-cell model: clinical challenges and opportunities. Lancet Oncol. 2012; 13:e83–89. 10.1016/S1470-2045(11)70257-1 22300863

[5] Li L, Liu Y, Guo Y, Liu B, Zhao Y, Li P, Song F, Zheng H, Yu J, Song T, Niu R, Li Q, Wang XW, et al. Regulatory MiR-148a-ACVR1/BMP circuit defines a cancer stem cell-like aggressive subtype of hepatocellular carcinoma. Hepatology. 2015; 61:574–84. 10.1002/hep.27543 25271001 PMC6311417

[6] Lacoste B, Raymond VA, Cassim S, Lapierre P, Bilodeau M. Highly tumorigenic hepatocellular carcinoma cell line with cancer stem cell-like properties. PLoS One. 2017; 12:e0171215. 10.1371/journal.pone.0171215 28152020 PMC5289561

[7] Visvader JE, Lindeman GJ. Cancer stem cells: current status and evolving complexities. Cell Stem Cell. 2012; 10:717–28. 10.1016/j.stem.2012.05.007 22704512

[8] Bhan A, Mandal SS. Long noncoding RNAs: emerging stars in gene regulation, epigenetics and human disease. ChemMedChem. 2014; 9:1932–56. 10.1002/cmdc.201300534 24677606

[9] Qu S, Yang X, Li X, Wang J, Gao Y, Shang R, Sun W, Dou K, Li H. Circular RNA: A new star of noncoding RNAs. Cancer Lett. 2015; 365:141–48. 10.1016/j.canlet.2015.06.003 26052092

[10] Yuan JH, Yang F, Wang F, Ma JZ, Guo YJ, Tao QF, Liu F, Pan W, Wang TT, Zhou CC, Wang SB, Wang YZ, Yang Y, et al. A long noncoding RNA activated by TGF-β promotes the invasion-metastasis cascade in hepatocellular carcinoma. Cancer Cell. 2014; 25:666–81. 10.1016/j.ccr.2014.03.010 24768205

[11] Hirata H, Hinoda Y, Shahryari V, Deng G, Nakajima K, Tabatabai ZL, Ishii N, Dahiya R. Long Noncoding RNA MALAT1 Promotes Aggressive Renal Cell Carcinoma through Ezh2 and Interacts with miR-205. Cancer Res. 2015; 75:1322–31. 10.1158/0008-5472.CAN-14-2931 25600645 PMC5884967

[12] Liu B, Sun L, Liu Q, Gong C, Yao Y, Lv X, Lin L, Yao H, Su F, Li D, Zeng M, Song E. A cytoplasmic NF-κB interacting long noncoding RNA blocks IκB phosphorylation and suppresses breast cancer metastasis. Cancer Cell. 2015; 27:370–81. 10.1016/j.ccell.2015.02.004 25759022

[13] Chen Z, Luo Y, Yang W, Ding L, Wang J, Tu J, Geng B, Cui Q, Yang J. Comparison Analysis of Dysregulated LncRNA Profile in Mouse Plasma and Liver after Hepatic Ischemia/Reperfusion Injury. PLoS One. 2015; 10:e0133462. 10.1371/journal.pone.0133462 26221732 PMC4519261

[14] Gupta RA, Shah N, Wang KC, Kim J, Horlings HM, Wong DJ, Tsai MC, Hung T, Argani P, Rinn JL, Wang Y, Brzoska P, Kong B, et al. Long non-coding RNA HOTAIR reprograms chromatin state to promote cancer metastasis. Nature. 2010; 464:1071–76. 10.1038/nature08975 20393566 PMC3049919

[15] Ma MZ, Chu BF, Zhang Y, Weng MZ, Qin YY, Gong W, Quan ZW. Long non-coding RNA CCAT1 promotes gallbladder cancer development via negative modulation of miRNA-218-5p. Cell Death Dis. 2015; 6:e1583. 10.1038/cddis.2014.541 25569100 PMC4669740

[16] Shi Y, Liu Y, Wang J, Jie D, Yun T, Li W, Yan L, Wang K, Feng J. Downregulated long noncoding RNA BANCR promotes the proliferation of colorectal cancer cells via downregualtion of p21 expression. PLoS One. 2015; 10:e0122679. 10.1371/journal.pone.0122679 25928067 PMC4415816

[17] He Y, Meng XM, Huang C, Wu BM, Zhang L, Lv XW, Li J. Long noncoding RNAs: novel insights into hepatocelluar carcinoma. Cancer Lett. 2014; 344:20–27. 10.1016/j.canlet.2013.10.021 24183851

[18] Lee S, Kopp F, Chang TC, Sataluri A, Chen B, Sivakumar S, Yu H, Xie Y, Mendell JT. Noncoding RNA NORAD Regulates Genomic Stability by Sequestering PUMILIO Proteins. Cell. 2016; 164:69–80. 10.1016/j.cell.2015.12.017 26724866 PMC4715682

[19] Liu W, Ma C, Yang B, Yin C, Zhang B, Xiao Y. LncRNA Gm15290 sponges miR-27b to promote PPARγ-induced fat deposition and contribute to body weight gain in mice. Biochem Biophys Res Commun. 2017; 493:1168–75. 10.1016/j.bbrc.2017.09.114 28943435

[20] Yu SY, Dong B, Zhou SH, Tang L. LncRNA UCA1 modulates cardiomyocyte apoptosis by targeting miR-143 in myocardial ischemia-reperfusion injury. Int J Cardiol. 2017; 247:31. 10.1016/j.ijcard.2017.05.055 28916071

[21] Li Z, Rana TM. Decoding the noncoding: prospective of lncRNA-mediated innate immune regulation. RNA Biol. 2014; 11:979–85. 10.4161/rna.29937 25482890 PMC4615744

[22] Mirza AH, Kaur S, Brorsson CA, Pociot F. Effects of GWAS-associated genetic variants on lncRNAs within IBD and T1D candidate loci. PLoS One. 2014; 9:e105723. 10.1371/journal.pone.0105723 25144376 PMC4140826

[23] Yan B, Yao J, Liu JY, Li XM, Wang XQ, Li YJ, Tao ZF, Song YC, Chen Q, Jiang Q. lncRNA-MIAT regulates microvascular dysfunction by functioning as a competing endogenous RNA. Circ Res. 2015; 116:1143–56. 10.1161/CIRCRESAHA.116.305510 25587098

[24] Wilusz JE, Sharp PA. Molecular biology. A circuitous route to noncoding RNA. Science. 2013; 340:440–41. 10.1126/science.1238522 23620042 PMC4063205

[25] Li H, Yang J, Wei X, Song C, Dong D, Huang Y, Lan X, Plath M, Lei C, Ma Y, Qi X, Bai Y, Chen H. CircFUT10 reduces proliferation and facilitates differentiation of myoblasts by sponging miR-133a. J Cell Physiol. 2018; 233:4643–51. 10.1002/jcp.26230 29044517

[26] Hansen TB, Jensen TI, Clausen BH, Bramsen JB, Finsen B, Damgaard CK, Kjems J. Natural RNA circles function as efficient microRNA sponges. Nature. 2013; 495:384–88. 10.1038/nature11993 23446346

[27] Deng T, Yang L, Zheng Z, Li Y, Ren W, Wu C, Guo L. Calcitonin gene-related peptide induces IL-6 expression in RAW264.7 macrophages mediated by mmu_circRNA_007893. Mol Med Rep. 2017; 16:9367–74. 10.3892/mmr.2017.7779 29039515 PMC5779990

[28] Kong Z, Wan X, Zhang Y, Zhang P, Zhang Y, Zhang X, Qi X, Wu H, Huang J, Li Y. Androgen-responsive circular RNA circSMARCA5 is up-regulated and promotes cell proliferation in prostate cancer. Biochem Biophys Res Commun. 2017; 493:1217–23. 10.1016/j.bbrc.2017.07.162 28765045

[29] Zhou M, Zhao H, Wang Z, Cheng L, Yang L, Shi H, Yang H, Sun J. Identification and validation of potential prognostic lncRNA biomarkers for predicting survival in patients with multiple myeloma. J Exp Clin Cancer Res. 2015; 34:102–102. 10.1186/s13046-015-0219-5 26362431 PMC4567800

[30] Cui X, Niu W, Kong L, He M, Jiang K, Chen S, Zhong A, Li W, Lu J, Zhang L. hsa_circRNA_103636: potential novel diagnostic and therapeutic biomarker in Major depressive disorder. Biomark Med. 2016; 10:943–52. 10.2217/bmm-2016-0130 27404501

[31] Li C, Du Y, Yang Z, He L, Wang Y, Hao L, Ding M, Yan R, Wang J, Fan Z. GALNT1-Mediated Glycosylation and Activation of Sonic Hedgehog Signaling Maintains the Self-Renewal and Tumor-Initiating Capacity of Bladder Cancer Stem Cells. Cancer Res. 2016; 76:1273–83. 10.1158/0008-5472.CAN-15-2309 26676748

[32] Pastrana E, Silva-Vargas V, Doetsch F. Eyes wide open: a critical review of sphere-formation as an assay for stem cells. Cell Stem Cell. 2011; 8:486–98. 10.1016/j.stem.2011.04.007 21549325 PMC3633588

[33] Lee J, Kotliarova S, Kotliarov Y, Li A, Su Q, Donin NM, Pastorino S, Purow BW, Christopher N, Zhang W, Park JK, Fine HA. Tumor stem cells derived from glioblastomas cultured in bFGF and EGF more closely mirror the phenotype and genotype of primary tumors than do serum-cultured cell lines. Cancer Cell. 2006; 9:391–403. 10.1016/j.ccr.2006.03.030 16697959

[34] Roessler S, Jia HL, Budhu A, Forgues M, Ye QH, Lee JS, Thorgeirsson SS, Sun Z, Tang ZY, Qin LX, Wang XW. A unique metastasis gene signature enables prediction of tumor relapse in early-stage hepatocellular carcinoma patients. Cancer Res. 2010; 70:10202–12. 10.1158/0008-5472.CAN-10-2607 21159642 PMC3064515

[35] Paraskevopoulou MD, Georgakilas G, Kostoulas N, Reczko M, Maragkakis M, Dalamagas TM, Hatzigeorgiou AG. DIANA-LncBase: experimentally verified and computationally predicted microRNA targets on long non-coding RNAs. Nucleic Acids Res. 2013; 41:D239–45. 10.1093/nar/gks1246 23193281 PMC3531175

[36] Zhang XO, Wang HB, Zhang Y, Lu X, Chen LL, Yang L. Complementary sequence-mediated exon circularization. Cell. 2014; 159:134–47. 10.1016/j.cell.2014.09.001 25242744

[37] Mao X, Cai T, Olyarchuk JG, Wei L. Automated genome annotation and pathway identification using the KEGG Orthology (KO) as a controlled vocabulary. Bioinformatics. 2005; 21:3787–93. 10.1093/bioinformatics/bti430 15817693

[38] Kanehisa M, Araki M, Goto S, Hattori M, Hirakawa M, Itoh M, Katayama T, Kawashima S, Okuda S, Tokimatsu T, Yamanishi Y. KEGG for linking genomes to life and the environment. Nucleic Acids Res. 2008; 36:D480–84. 10.1093/nar/gkm882 18077471 PMC2238879

[39] Ho PL, Kurtova A, Chan KS. Normal and neoplastic urothelial stem cells: getting to the root of the problem. Nat Rev Urol. 2012; 9:583–94. 10.1038/nrurol.2012.142 22890301 PMC3468664

[40] Uchiyama N, Dougan DR, Lawson JD, Kimura H, Matsumoto SI, Tanaka Y, Kawamoto T. Identification of AHCY inhibitors using novel high-throughput mass spectrometry. Biochem Biophys Res Commun. 2017; 491:1–7. 10.1016/j.bbrc.2017.05.107 28533090

[41] Park SJ, Kong HK, Kim YS, Lee YS, Park JH. Inhibition of S-adenosylhomocysteine hydrolase decreases cell mobility and cell proliferation through cell cycle arrest. Am J Cancer Res. 2015; 5:2127–38. 26328244 PMC4548325

[42] Lindquist D, Kvarnbrink S, Henriksson R, Hedman H. LRIG and cancer prognosis. Acta Oncol. 2014; 53:1135–42. 10.3109/0284186X.2014.953258 25180912 PMC4438349

[43] Wang Y, Poulin EJ, Coffey RJ. LRIG1 is a triple threat: ERBB negative regulator, intestinal stem cell marker and tumour suppressor. Br J Cancer. 2013; 108:1765–70. 10.1038/bjc.2013.138 23558895 PMC3658528

[44] Guo G, Sun X, Chen C, Wu S, Huang P, Li Z, Dean M, Huang Y, Jia W, Zhou Q, Tang A, Yang Z, Li X, et al. Whole-genome and whole-exome sequencing of bladder cancer identifies frequent alterations in genes involved in sister chromatid cohesion and segregation. Nat Genet. 2013; 45:1459–63. 10.1038/ng.2798 24121792 PMC7512009

[45] Tao T, Yang X, Zheng J, Feng D, Qin Q, Shi X, Wang Q, Zhao C, Peng Z, Liu H, Jiang WG, He J. PDZK1 inhibits the development and progression of renal cell carcinoma by suppression of SHP-1 phosphorylation. Oncogene. 2017; 36:6119–31. 10.1038/onc.2017.199 28692056

[46] Su H, Tao T, Yang Z, Kang X, Zhang X, Kang D, Wu S, Li C. Circular RNA cTFRC acts as the sponge of MicroRNA-107 to promote bladder carcinoma progression. Mol Cancer. 2019; 18:27. 10.1186/s12943-019-0951-0 30782157 PMC6379985

[47] Chandrashekar DS, Bashel B, Balasubramanya SA, Creighton CJ, Ponce-Rodriguez I, Chakravarthi BV, Varambally S. UALCAN: A Portal for Facilitating Tumor Subgroup Gene Expression and Survival Analyses. Neoplasia. 2017; 19:649–58. 10.1016/j.neo.2017.05.002 28732212 PMC5516091

