# Supplementary figures and images for: Cancer stem cell-specific expression profiles reveal emerging bladder cancer biomarkers and identify circRNA_103809 as an important regulator in bladder cancer
Source: Aging (Albany NY). 2020 Feb 17;12(4):3354–70. doi: 10.18632/aging.102816 (PMC7066924; doi:10.18632/aging.102816)

SUPPLEMENTARY FIGURE

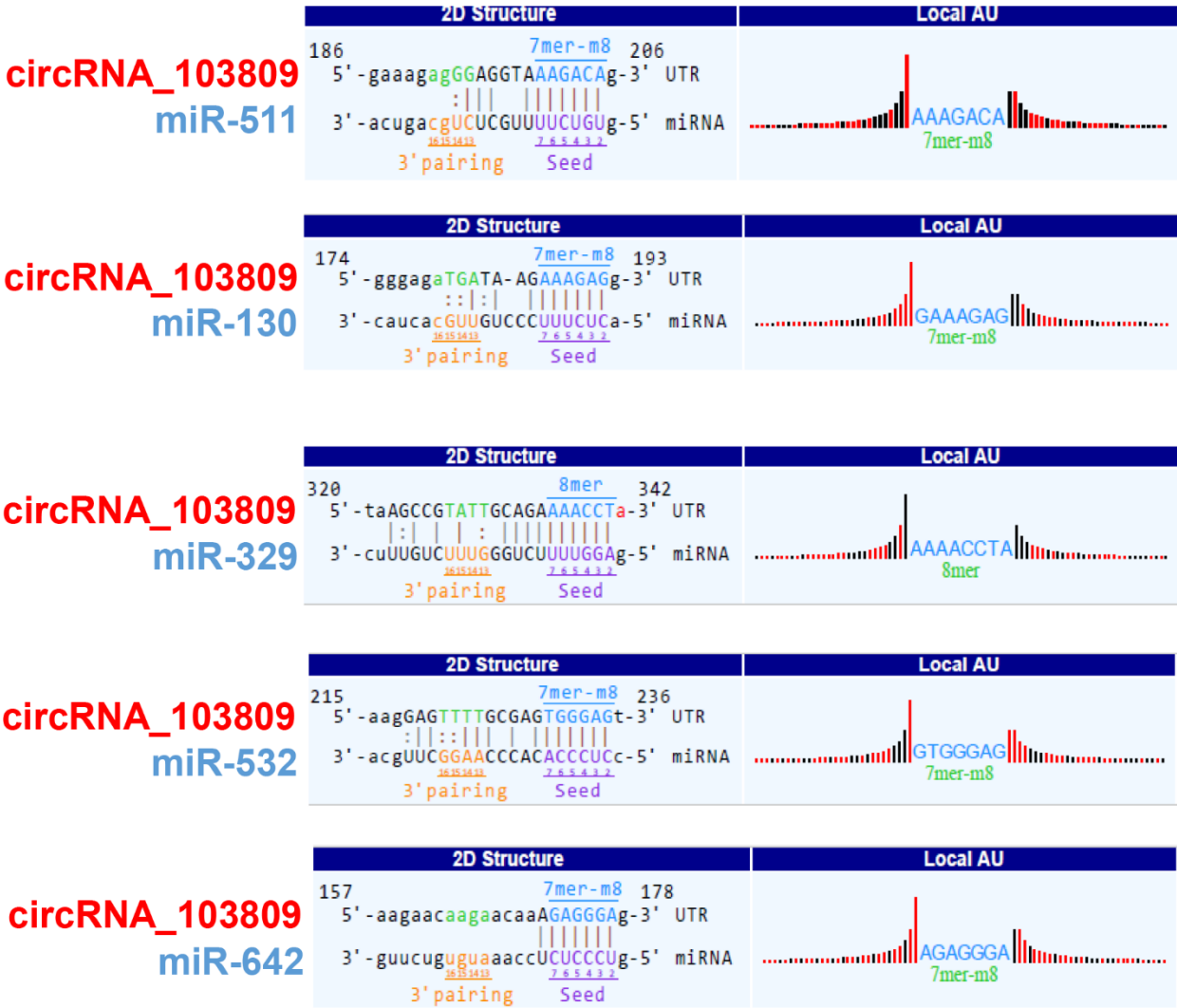

Supplementary Figure 1. Binding sites of miRNAs in 3'UTR of circRNA\_103809.

Supplement: Supplementary Figure 1 [file aging-12-102816-s002..pdf]
