# Supplementary material for: Cancer stem cell-specific expression profiles reveal emerging bladder cancer biomarkers and identify circRNA_103809 as an important regulator in bladder cancer
Source: Aging (Albany NY). 2020 Feb 17;12(4):3354–70. doi: 10.18632/aging.102816 (PMC7066924; doi:10.18632/aging.102816)
Supplement: Supplementary Table 1 [file aging-12-102816-s001..pdf]

## SUPPLEMENTARY TABLE

**Supplementary Table 1. Primers for qRT-PCR analysis.**

| Target gene | Forward primer | Reverse primer | RT primer |
|---|---|---|---|
| XAGE5 | CTGGTTGGGCCTATGCTTGAG | TCCGCATTCATCCCCAGTCT | |
| COX7B2 | CCAGAAATGCACTAAGCAGTCT | ACCCATGTAGCAACACAGAAAG | |
| MAGEB2 | GCTGCGGGTGTTTCATCCA | TGGTTAGAGGATCTTCGCTTGG | |
| BHMT | TGCTGGAGAGATTGTGATTGGA | CTTGTCTTCACTCGCATAGAAGG | |
| PROM1 | AGTCGGAAACTGGCAGATAGC | GGTAGTGTTGTACTGGGCCAAT | |
| SLCO1B3 | TGGAGCAACAGTACGGTCAG | TGCTTTCGCAGATTAGAGGGAA | |
| RP11-332K15.1 | ACTCTCGGGATCCTGGACCT | AGTCCTCAGTGCTTGCGACA | |
| XX-CR54.1 | TGTACTCATCAGCACGGCATAAGG | ATCAGAAGGCAACACTCAGGACTG | |
| BC038578 | AGCCAGCGAGACCACGAACC | TAAGGTGGCGTGTCTGGAGTCTG | |
| LOC389023 | CGTGTCTGAGATTGTGGCCTGAG | TAGGAGTTCCACCGACGTGACC | |
| SSTR5-AS1 | GTGTTCCTCCATGAAGAGCAGAGC | GCACGCAGCACACTCCTTCC | |
| FAM99A | GGCCTGGCTCACTCCGTGTC | TCCTCTGGCTCTCCTTCATTCTGC | |
| hsa_circRNA_103809 | TCGAGACCTCTGTCAGCGAG | GCCAATACTCCCACTCGCAA | |
| hsa_circRNA_101368 | AAGACTTGAGGCGAATGG | GCACAGGTGAATAGACTTCT | |
| hsa_circRNA_102399 | GTGCAGGGTCCGAGGTATT | GCCGTCGTAATACTGCCTGGT | |
| hsa_circRNA_000639 | ATGCCCACAGCTTTCCAA | TCTCCTTTCTCCCTCTACATT | |
| hsa_circRNA_001547 | ACATACCCGTTGGCTCTC | CAGGGTCATCCACAATCAG | |
| hsa_circRNA_400010 | GTATAGGATGACTCACTGACA | GCACTCAACAATCGTTAGC | |
| miR-532 | GCCTCCCACACCCAAGG | AGTGCAGGGTCCGAGGTATT | GTCGTATCCAGTGCAGGGTCCGAGGTATTCGCACTGGATACGACTGCAAG |
| miR-130 | CGCGACTCTTTCCCTGTTG | AGTGCAGGGTCCGAGGTATT | GTCGTATCCAGTGCAGGGTCCGAGGTATTCGCACTGGATACGACGTAGTG |
| miR-642 | GCGGTCCCTCTCCAAATGT | AGTGCAGGGTCCGAGGTATT | GTCGTATCCAGTGCAGGGTCCGAGGTATTCGCACTGGATACGACCAAGAC |
| miR-329 | GCGGAGGTTTTCTGGGTTTC | AGTGCAGGGTCCGAGGTATT | GTCGTATCCAGTGCAGGGTCCGAGGTATTCGCACTGGATACGACGAAACA |
| miR-511 | CGCGGTGTCTTTTGCTCTG | AGTGCAGGGTCCGAGGTATT | GTCGTATCCAGTGCAGGGTCCGAGGTATTCGCACTGGATACGACTGACTG |
